# FDA Modernization Act 2.0: transitioning beyond animal models with human cells, organoids, and AI/ML-based approaches

**DOI:** 10.1172/JCI175824

**Published:** 2023-11-01

**Authors:** Peter-James H. Zushin, Souhrid Mukherjee, Joseph C. Wu

**Affiliations:** 1 Stanford Cardiovascular Institute and; 2 Department of Medicine (Division of Cardiology), Stanford University, Stanford, California, USA.; 3 Greenstone Biosciences, Palo Alto, California, USA.

## Introduction

The discovery and generation of effective therapeutics to combat disease lies at the heart of biomedical research. Preclinical studies form the foundation of potential disease treatments, guiding their journey from scientific discovery to impactful patient outcomes. However, over the past two decades, preclinical research has been frequently plagued by the failure to replicate consistent results, costing an estimated $28 billion USD per year (1). Potential therapeutics from preclinical studies entering phase I trials only had a 10.4% approval rate between 2003 and 2014 (2) and an even lower 6% to 7% rate between 2011 and 2017 (3). The disappointing reality of promising preclinical findings that fail to translate into effective therapies has raised serious concerns within the scientific community (4). The cause of this failure is potentially elucidated in a 2015 retrospective analysis of four large biotech companies that showed the most common causes of termination in phase I and II clinical trials since 2003 are the lack of efficacy (60% of all trials) and toxicity (30%) (5). Given these insights and the emergence of advanced technologies that enable large-cohort, in vitro human testing, a pressing need to reassess our approaches to studying human diseases exists. Such changes are vital to facilitating the development of lifesaving therapeutics that can extend both health span and life span by more efficiently translating from the bench to the bedside.

Since the passage of the Federal Food, Drug, and Cosmetic Act in 1938, the FDA has required the use of animal models for the initial scientific and safety validation of preclinical therapeutics in order to proceed to phase 1 clinical trials. The FDA accepts a range of animal models so long as they reflect the disease or condition being targeted. Traditionally, the vast majority of biomedical research has relied on rodent models primarily because of rodents’ short life span, high fecundity, and ease of genetic modification (6). Although animal models have helped us achieve significant strides in our understanding of fundamental biology, researchers increasingly recognize the fundamental species differences between animal models and humans that have substantial pharmacogenomic implications. These disparities can confound results and likely contribute greatly to the high failure of promising therapeutics advancing to later stage clinical trials.

## Interrogating why clinical trials often go awry

Pharmacogenomics is the study of how genetics affects individual responses to drugs and accounts for the substantial variation in drug sensitivity of different individuals (7). Pharmacogenomics can also markedly differ between humans and other animal models, resulting in vastly different results when comparing the efficacy and toxicity of a new therapeutic. For example, the pharmacokinetics of mice and humans differ in terms of drug absorption, distribution, metabolism, and excretion (ADME) owing to factors that include blood-flow rates, tissue specificity, organ sizes, and nonconserved biological processes. Enzymes such as cytochrome P450 that are involved in drug metabolism vary in number, leading to differences in how drugs are broken down and cleared from the body. When the pharmacogenomics of model organisms extensively differ from that of humans, promising drug candidates can fail or be withdrawn after reaching the market. One such example was the phase I trial failure of theralizumab, an anti-CD28 monoclonal antibody intended for use in arthritis and B cell lymphocytic leukemia (B-CLL). Preclinical experiments showed great efficacy in a BALB/c mouse model of B-CLL, but in humans, a low dose (1/500th of the safe dose in mice) induced a massive cytokine storm, resulting in organ failure and hospitalization (8). Due to the distinct variations in how genes influence drug responses between mice and humans, it is essential to prioritize research directions that are based on the most accurate science.

**Figure 1:**
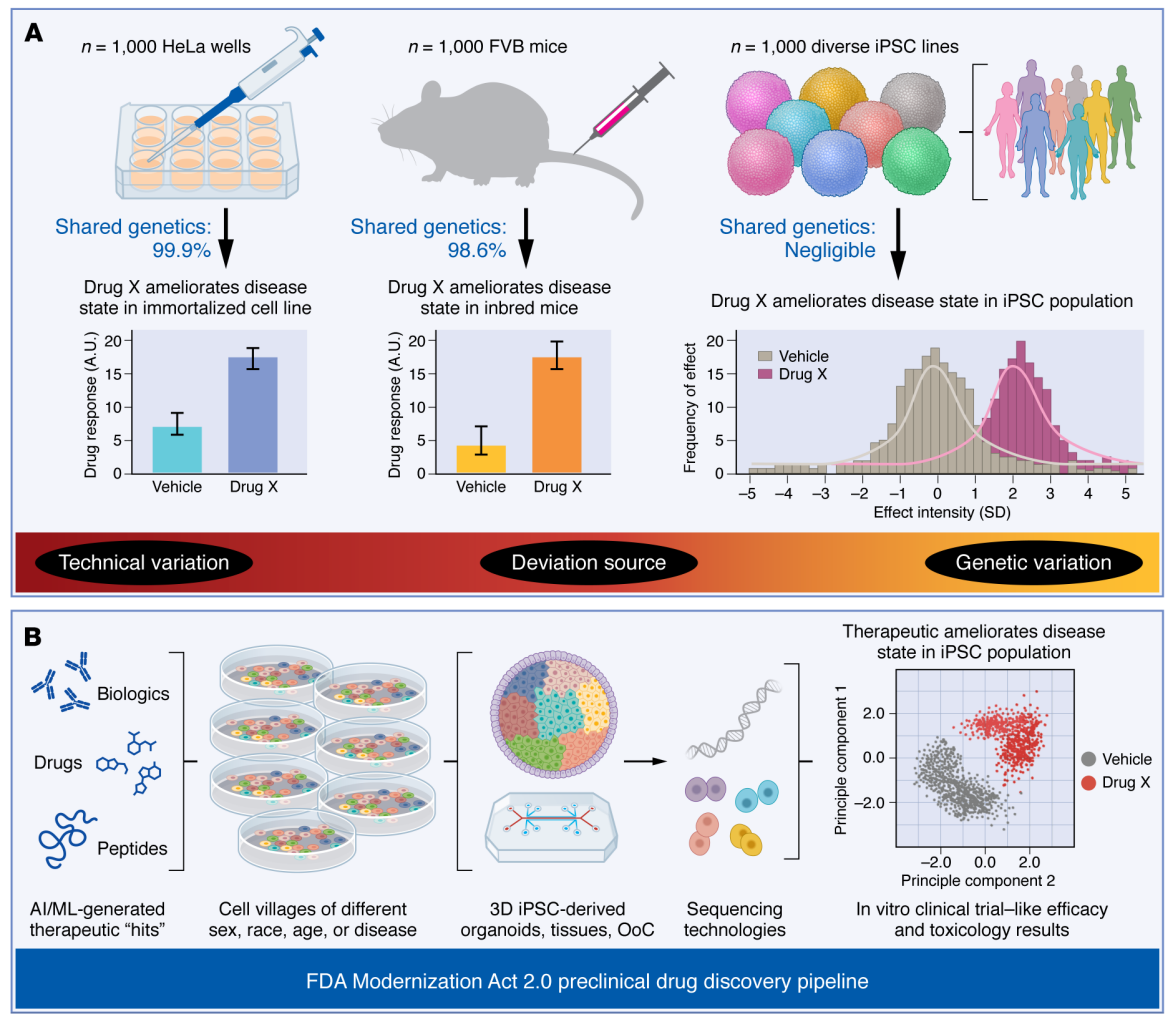
Lack of genetic diversity and pharmacogenomic differences between model animals and humans lead to the high termination rate of phase I and II clinical trials. (**A**) Immortalized cell lines such as HeLa cells and inbred rodent models are commonly used as the gold standard of preclinical validation due to their ease of use. However, tissue cultures use immortalized cell lines with 99.9% shared genetics on average and rodent experiments use inbred strains with 98.6% shared genetics on average. Data from technical replicates of therapeutic agents can have positive results with low standard deviations that may be misleading. In contrast, testing therapeutic agents on patient-specific iPSCs more accurately represents the full genetic and pharmacogenomic diversity of human populations. Here, experimental results would reflect the effects of therapeutic agents in responders and nonresponders across a large cohort of individuals (rather than in a single immortalized cell line or a chosen rodent strain), hence providing more reliable safety and efficacy data prior to proceeding to clinical trials. (**B**) Innovative techniques for cultivating multiple iPSC lines together and segregating their transcriptomic and genetic signals have given rise to the concept of “cell villages.” These pooled populations are then differentiated into various cell types and incorporated into 3D models such as organoids or organs-on-chips, closely mimicking the human in vivo environment. These cell village tissues can then be subjected to therapeutic agents identified from AI/ML models, and the application of single-cell technologies allows for elucidation of each cell line’s distinct gene expression response to the therapeutic intervention. This transformative approach of clinical-trial-in-a-dish holds immense potential for enhancing our understanding of drug safety and efficacy, ultimately increasing the likelihood of therapeutic success in early clinical trials.

The inbred nature of animals used in rodent models, as opposed to the considerable genetic variability of humans, plays a substantial role in the poor translation rates of animal model–based therapeutics (Figure 1A). Humans have a vast genetic diversity that leads to variation in drug metabolism, drug targets, and drug interactions among individuals within any given population. Many human diseases stem from the intricate interplay of genetics and environmental influences that accumulate over a lifetime. This accumulation of inputs, stressors, and life experiences contributes to the unique ways that diseases manifest and how they respond to treatments in each individual. Mouse models cannot accurately simulate the complexities of disease presentation in humans (9).

As few as 1 in 25 people are optimal responders to common medications (10), which leads to questions as to whether clinical trials in their current form are actually testing enough individuals to characterize the compound in a broad swath of humanity. In contrast, each mouse shares roughly 98.6% of its genome with others of the same strain, meaning they can effectively be treated as clones (11). Therefore, data points obtained from individual animals are more akin to technical replicates (similar to an immortalized cell-line experiment) rather than biological replicates due to their lack of genetic diversity (Figure 1A). Placing less emphasis on animal model results and more on human cell-based models that more accurately reflect the diversity of human genetics may guide the development of treatments that are more reliable and successful in real-world applications.

Recently, legislators and the FDA recognized the issues inherent in animal models and took steps to address it. On December 29, 2022, President Biden signed the FDA Modernization Act 2.0 into law. This comprehensive legislation permits the utilization of specific alternatives to animal testing, including cell-based assays, such as human induced pluripotent stem cells (iPSCs), organoids and organs-on-chips (OoCs), and advanced artificial intelligence (AI) methods, such as generative adversarial networks (GANs) and language models (12). AI/machine learning (ML) methods have been leveraged over the past several years to predict drug toxicity, metabolism, and other ADME properties, and recently, many AI pipelines have been created to evaluate off-target effects for drugs incorporating structural biology and multiomics data as well (13). Also, generative AI is being used extensively to augment sparse real-world data and even design in silico clinical trials using synthetic digital twin technology (14). These alternatives can now be used to seek FDA exemptions for assessing drug safety and effectiveness during the preclinical phase. This legislation signifies a major shift that allows preclinical studies to adopt methods that may more accurately predict human responses based on the latest scientific advances rather than being solely dependent on increasingly outdated animal testing.

## Pioneering technologies to bridge preclinical to clinical translation

The incorporation of human-relevant in vitro models, such as human iPSCs (15), has gained traction as a robust substitute for animal testing in preclinical drug development. These models more reliably mirror human physiology, disease characteristics, and pharmacogenomics. Consequently, they offer a potent alternative for enhancing the accuracy and relevance of preclinical research, leading to better translation from the bench to clinical trials. iPSCs are created from easily collected somatic cell sources, such as skin fibroblasts, leukocytes, or urine, after they have been reprogrammed using the four Yamanaka factors (OCT4, SOX2, KLF4, and cMYC). Since the protocol publication in 2006, the generation of iPSCs has become a routine and manageable process, evident from the establishment of numerous biobanks globally, including one here at the Stanford Cardiovascular Institute. In partnership with clinicians, we currently maintain a repository of more than 2,000 iPSC lines (as of Fall 2023) sourced from sexually and ethnically diverse individuals — both healthy and those with mutations linked to common and rare human diseases. By reprogramming cells from patients with specific genetic mutations, scientists can create cellular models that recapitulate disease characteristics to improve our understanding of disease mechanisms and drug testing.

Biotechnology has undergone rapid growth in the past 15 years, enabling a field such as bioengineering to utilize iPSCs to create more innovative approaches for use in personalized medicine. A good example of this is the 2D-to-3D transition of the culture of iPSCs, producing a more physiologically robust system to perform translational wet lab experiments. Novel technologies such as iPSC-derived organoids, engineered heart tissues (15), neural tissue constructs, and OoCs (16), generate multicellular isogenic tissue mimics to model vital aspects of that tissue’s functions. OoCs are exquisitely designed as well as highly specific to their application, containing media channels and cell chambers formed by layers of polydimethylsiloxane (PDMS) created with photolithography and isoporous membranes. These “chips,” or microphysiological devices, can mix and match cell types, creating architectures and zones of influence similar to those of native tissues with the ability to have multiple organs within one device to simulate organ crosstalk (17). While these 3D technologies are not yet suited to running of clinical trials in a chip or dish, these innovative bioengineering approaches showcase the potential of iPSCs in bridging the gap between preclinical science and clinical trials by creating more physiologically relevant in vitro assays.

The potential of human cell models and clinical-trial-in-a-dish methodologies to improve drug discovery outcomes in preclinical research is immense; however, two roadblocks exist that are standing in the way of their wider use. First, the genetic diversity of humanity is difficult to work with, as increasing the numbers of individual cell lines tested means an investment in research time and space to accommodate them. Second, using bioengineered 3D models that properly replicate tissue-level aspects of biology often results in the lack of ability to scale up into cell numbers that are viable for higher-throughput methods that allow more lines to be tested. To address this, iPSC-based methods of preclinical research must increase in size to be able to better represent a meaningful cohort of diverse individuals while maintaining cost effectiveness of experiments that are able to be carried out in an academic research environment. Recent advances in single-cell technologies have opened the door for these large-scale experiments by using pooled cell lines or “cell villages.” These cell villages are made feasible by the ability of whole-genome sequencing to capture variations in small sequence reads, which allows each line to be barcoded, making it possible for single-cell sequencing methodologies such as RNA and ATAC sequencing to be assigned back to the original donor (18). Utilizing this innovative method not only allows researchers to significantly increase their experimental throughput, but also enables the simultaneous analysis of gene expression patterns across a multitude of diverse genetic profiles, bringing the field a step closer to conducting preclinical efficacy and toxicology trials on a scale encompassing hundreds, or even thousands, of human iPSCs (Figure 1B).

## Conclusion

The discovery and development of effective therapeutics to combat diseases is a cornerstone of biomedical research. Preclinical studies serve as a crucial gateway, bridging scientific discovery with tangible patient outcomes, yet still often fail to provide positive results. The FDA Modernization Act 2.0 has paved the way for alternative methods to bolster the preclinical data pipeline, aiming to reduce the dependence on animal models that have frequently resulted in therapeutic dead ends. Among these new alternatives, iPSCs in particular have gained prominence for their potential to offer more physiologically relevant insights into disease mechanisms and drug responses. When paired with emerging bioengineered 3D techniques and sequencing technologies, iPSCs currently represent the best hope for improving our preclinical-to-clinical trial pipeline for new therapeutics.

## Version 1 11/01/2023

Electronic publication

## References

[1] Freedman LP (2015). The economics of reproducibility in preclinical research. PLoS Biol.

[2] Hay M (2014). Clinical development success rates for investigational drugs. Nat Biotechnol.

[3] Dowden H, Munro J (2019). Trends in clinical success rates and therapeutic focus. Nat Rev Drug Discov.

[4] Prinz F (2011). Believe it or not: how much can we rely on published data on potential drug targets?. Nat Rev Drug Discov.

[5] Waring MJ (2015). An analysis of the attrition of drug candidates from four major pharmaceutical companies. Nat Rev Drug Discov.

[6] Yoshiki A (2022). Genetic quality: a complex issue for experimental study reproducibility. Transgenic Res.

[7] Becquemont L (2009). Pharmacogenomics of adverse drug reactions: practical applications and perspectives. Pharmacogenomics.

[8] Suntharalingam G (2006). Cytokine storm in a phase 1 trial of the anti-CD28 monoclonal antibody TGN1412. N Engl J Med.

[9] Zeggini E (2019). Translational genomics and precision medicine: Moving from the lab to the clinic. Science.

[10] Schork NJ (2015). Personalized medicine: Time for one-person trials. Nature.

[11] Beck JA (2000). Genealogies of mouse inbred strains. Nat Genet.

[12] Zhang A (2022). Shifting machine learning for healthcare from development to deployment and from models to data. Nat Biomed Eng.

[13] Rao MS (2019). Novel computational approach to predict off-target interactions for small molecules. Front Big Data.

[14] Arora A, Arora A (2022). Generative adversarial networks and synthetic patient data: current challenges and future perspectives. Future Healthc J.

[15] Cho S (2022). Challenges and opportunities for the next generation of cardiovascular tissue engineering. Nat Methods.

[16] Kim H (2022). Progress in multicellular human cardiac organoids for clinical applications. Cell Stem Cell.

[17] Low LA (2021). Organs-on-chips: into the next decade. Nat Rev Drug Discov.

[18] Wells MF (2023). Natural variation in gene expression and viral susceptibility revealed by neural progenitor cell villages. Cell Stem Cell.

